# Supplementary material for: Splice-Junction-Based Mapping of Alternative Isoforms in the Human Proteome
Source: Cell Rep. Author manuscript; Available in PMC 2020 Jan 15. (PMC6961840; doi:10.1016/j.celrep.2019.11.026)
Supplement: 3 [file NIHMS1546469-supplement-3.zip › DF2/PXD006675/PulmonaryArtery-58-Q13813-QEQIDNQYHSLLELGEK.pdf]

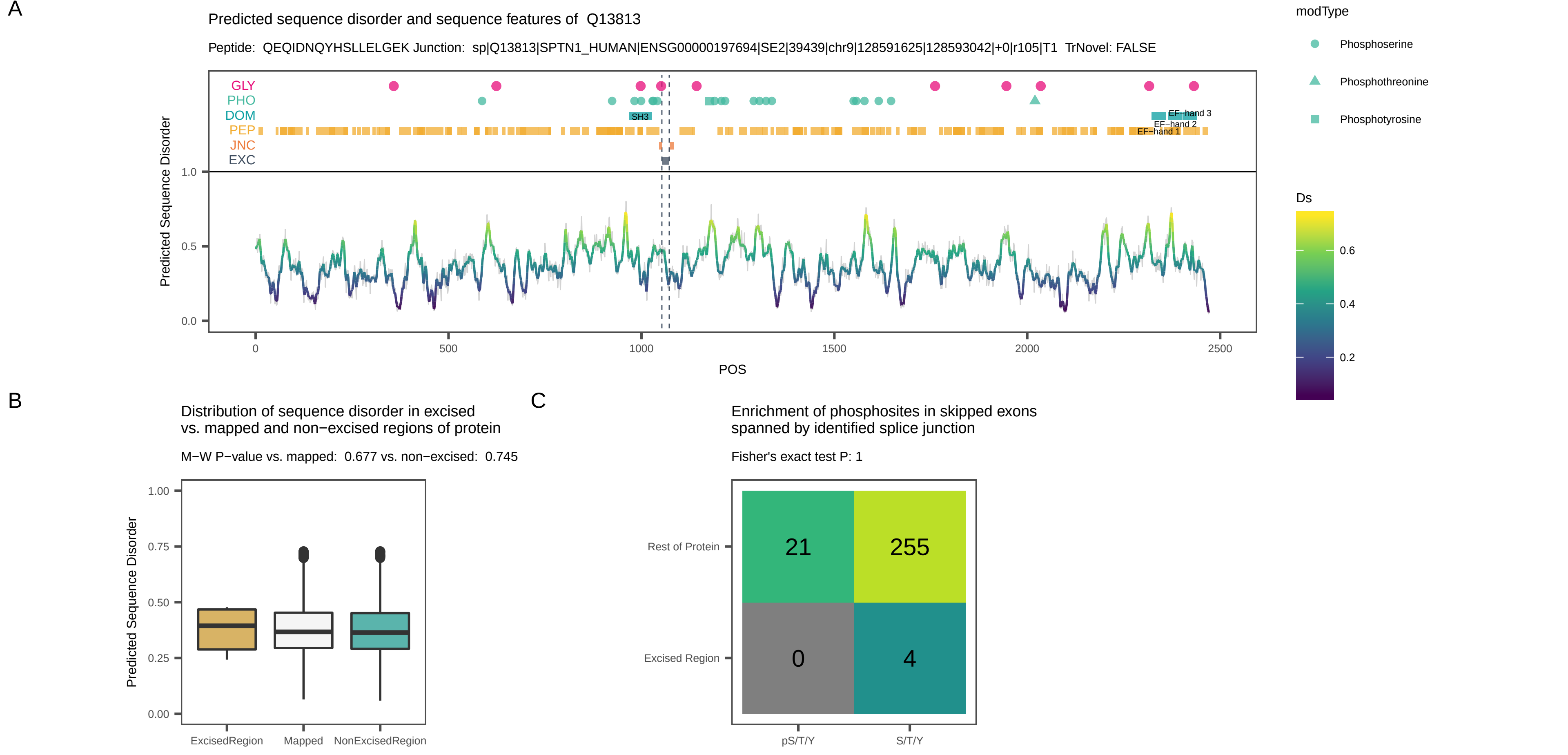
A
Predicted sequence disorder and sequence features of Q13813
Peptide: QEQIDNQYHSLLELGEK Junction: sp|Q13813|SPTN1_HUMAN|ENSG00000197694|SE2|39439|chr9|128591625|128593042|+0|r105|T1 TrNovel: FALSE
GLY
PHO
DOM
PEP
JNC
EXC
SH3
EF−hand 3
EF−hand 2
EF−hand 1
Predicted Sequence Disorder
1.0
0.5
0.0
0
500
1000
1500
2000
2500
POS
modType
Phosphoserine
Phosphothreonine
Phosphotyrosine
Ds
0.6
0.4
0.2
B
Distribution of sequence disorder in excised
vs. mapped and non−excised regions of protein
M−W P−value vs. mapped: 0.677 vs. non−excised: 0.745
Predicted Sequence Disorder
1.00
0.75
0.50
0.25
0.00
ExcisedRegion
Mapped
NonExcisedRegion
C
Enrichment of phosphosites in skipped exons
spanned by identified splice junction
Fisher's exact test P: 1
Rest of Protein
Excised Region
21
255
0
4
pS/T/Y
S/T/Y
